# Quantitation of the immunodominant 33-mer peptide from α-gliadin in wheat flours by liquid chromatography tandem mass spectrometry

**DOI:** 10.1038/srep45092

**Published:** 2017-03-22

**Authors:** Kathrin Schalk, Christina Lang, Herbert Wieser, Peter Koehler, Katharina Anne Scherf

**Affiliations:** 1 Deutsche Forschungsanstalt für Lebensmittelchemie, Leibniz Institut, Lise-Meitner-Straße 34, D-85354 Freising, Germany

## Abstract

Coeliac disease (CD) is triggered by the ingestion of gluten proteins from wheat, rye, and barley. The 33-mer peptide from α2-gliadin has frequently been described as the most important CD-immunogenic sequence within gluten. However, from more than 890 published amino acid sequences of α-gliadins, only 19 sequences contain the 33-mer. In order to make a precise assessment of the importance of the 33-mer, it is necessary to elucidate which wheat species and cultivars contain the peptide and at which concentrations. This paper presents the development of a stable isotope dilution assay followed by liquid chromatography tandem mass spectrometry to quantitate the 33-mer in flours of 23 hexaploid modern and 15 old common (bread) wheat as well as two spelt cultivars. All flours contained the 33-mer peptide at levels ranging from 91–603 μg/g flour. In contrast, the 33-mer was absent (<limit of detection) from tetra- and diploid species (durum wheat, emmer, einkorn), most likely because of the absence of the D-genome, which encodes α2-gliadins. Due to the presence of the 33-mer in all common wheat and spelt flours analysed here, the special focus in the literature on this most immunodominant peptide seems to be justified.

With a prevalence of about 1% in the Western population^1^, coeliac disease (CD) is one of the most common food hypersensitivities. This inflammatory disorder of the upper small intestine results in villous atrophy and consequently malabsorption of nutrients as well as extra- and intraintestinal symptoms. In genetically predisposed individuals, the precipitating factor of CD is the intake of storage proteins from wheat (gliadins, glutenins), barley (hordeins), rye (secalins), and possibly oats (avenins), which are called gluten in the field of CD^2^. CD patients need to follow a strict lifelong gluten-free diet to ensure mucosal healing and prevent complications.

All gluten protein fractions, namely the alcohol-soluble prolamins and the insoluble glutelins, contain CD-active epitopes^3^. The prolamin fraction is particularly rich in proline and glutamine and the numerous proline residues lead to a high resistance to complete proteolytic digestion by human gastric, pancreatic, and brushborder enzymes. Studies by Shan *et al*. (2002) showed that a large 33-mer peptide (LQLQPFPQPQLPYPQPQLPYPQPQLPYPQPQPF) from α2-gliadin (position in the amino acid sequence of α2-gliadin: 56–88) is resistant to cleavage by intestinal peptidases^4^,^5^. The 33-mer is widely called the most immunodominant gluten peptide^4^,^6^,^7^, because it contains three overlapping T-cell epitopes, namely PFPQPQLPY (DQ2.5-glia-α1a, one copy), PYPQPQLPY (DQ2.5-glia-α1b, two copies) and PQPQLPYPQ (DQ2.5-glia-α2, three copies)^3^, which result in the initiation of a strong immune response. As such, it plays an important role in the field of CD, e.g., as a model peptide to study CD mechanisms^7^,^8^,^9^ or the efficiency of gluten-degrading enzymes^10^,^11^,^12^. It consists of three heptamers (PQPQLPY) framed by a hexamer (PQPQPF) at the C-terminal end and a hexamer (LQLQPF) at the N-terminal end, which (positions 56–73) is also known to be CD-toxic from *in vivo* studies^9^. Arentz-Hansen *et al*. (2000) were the first to identify the 33-mer in α2-gliadin from the Norwegian common (bread) wheat (*Triticum aestivum*) cultivar (cv.) Mjølner (MJO). DNA-sequencing revealed the entire amino acid sequences of eleven α-gliadins (α1–α11) of this cultivar, but only α2-gliadin contained the 33-mer^13^. T-cell proliferation assays demonstrated that treatment of the 33-mer with tissue transglutaminase (TG2) resulted in a higher T-cell immune response after specific deamidation of the glutamine residues in positions 65 and 72^14^,^15^, followed by strong binding to HLA-DQ2^15^,^16^,^17^.

The high relevance of the 33-mer is reflected by the production of two monoclonal antibodies (A1 und G12) against the 33-mer peptide^18^. These are used in commercially available enzyme-linked immunosorbent assays (e.g., GlutenTox ELISA, Biomedal, Sevilla, Spain and AgraQuant^®^ ELISA Gluten G12, Romer Labs, Tulln, Austria) for the immunochemical quantitation of gluten in supposedly gluten-free foods^19^.

Due to its unique CD-epitope-rich structure, the 33-mer peptide plays an important role in the literature with 636 results for a search in the database ScienceDirect with “33 mer” and “celiac disease” as keywords (as of February 07, 2017). Although about 20 papers per year were published since 2000 and 53 for the year 2016, information about the quantities of 33-mer in different wheat species and cultivars is still missing. According to a BLAST search within 897 entries for α-gliadins from *Triticinae* in the UniProtKB database, the amino acid sequence of the 33-mer was found in only 16 protein sequences from *T. aestivum* and in three from *T. spelta* with an identity of 100% (as of February 07, 2017). Of these 19 sequences, only three have evidence at transcript level (Q9M4L6, Q1WA39 and A5JSA6) inferred from the three Chinese wheat cv. Gaocheng 8901, Zhongyou 9507, and Chinese Spring^20^,^21^, but only one (P18573) has evidence at protein level based on data of the Norwegian wheat cv. MJO. Taken together, the available data are insufficient to judge whether or not the 33-mer occurs frequently in different wheat species and cultivars.

Therefore, the aim of the present study was to develop a stable isotope dilution assay (SIDA) combined with targeted liquid chromatography tandem mass spectrometry (LC-MS/MS) for the quantitative determination of the 33-mer. The amount of 33-mer was determined in 57 samples of different wheat species from around the world (Table 1), including hexaploid common wheat (*T. aestivum*) and spelt (*T. aestivum* ssp. *spelta*), tetraploid durum wheat (*T. turgidum durum*) and emmer (*T. turgidum dicoccum*), and diploid einkorn (*T. monococcum*) to make a precise assessment of the importance of this peptide associated with CD.

## Results

### Development of a SIDA

**Figure 1:**
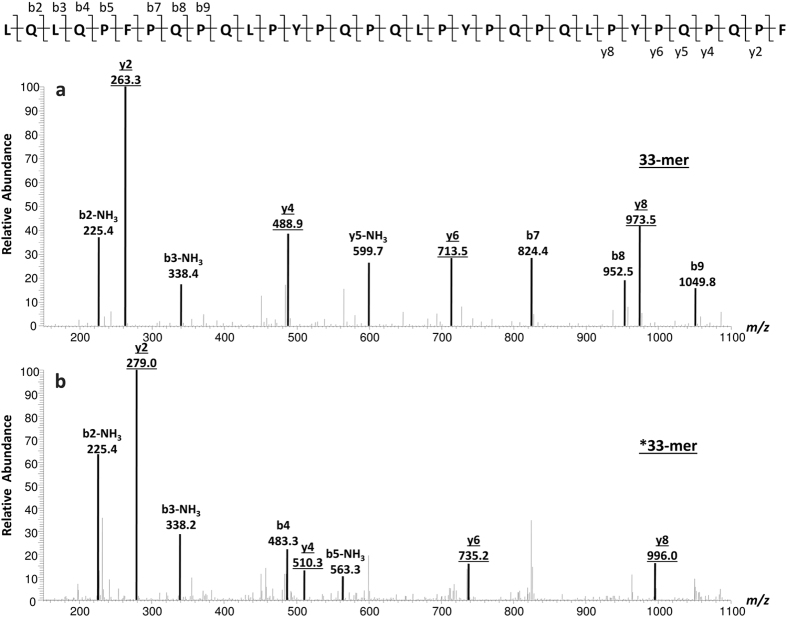
MS/MS product ion mass spectra of the 33-mer peptide (**a**) and the isotopically labelled *33-mer (**b**). The four most abundant product ions (underlined) were used for identification. The most abundant product ion (y2) was used for quantitation.

To develop a SIDA, a [^13^C_28_]- and [^15^N_4_]-labelled *33-mer peptide (LQLQP*FPQPQLPYPQPQLPYPQPQLPYPQ*PQ*P*F, with *F: L-[^13^C_9_][^15^N]-phenylalanine and *P: L-[^13^C_5_][^15^N]-proline, monoisotopic mass 3943.0) was used as isotopically labelled internal standard which differed by 32 mass units compared to the unlabelled analyte (33-mer, monoisotopic mass 3911.0). Based on the fragmentation pattern of the 33-mer (Fig. 1), the [^13^C]/[^15^N]-labelled amino acids were positioned in such a way that the label remained in the detected product ions. To define the most abundant transitions for multiple reaction monitoring (MRM), the 3+ and 4+ charge states of the 33-mer with *m/z* 1305.2 (3+) and *m/z* 979.0 (4+) and *33-mer standard with *m/z* 1316.0 (3+) and *m/z* 987.0 (4+) were totally fragmented. The 4+ charge state of the 33-mer was more abundant than the 3+ charge state state (ratio charge state (4+)/(3+) = 2/1) and, therefore, the most abundant MRM transition of the 4+ charged 33-mer analyte and isotopically labelled *33-mer standard was chosen for quantitation (quantifier for 33-mer *m/z* 979.0 → 263.3 and *33-mer *m/z* 987.0 → 279.2). The three MRM transitions (Table 2) following in intensity were used for qualification (qualifiers) as well as the MRM transitions of the 3+ charge state. The collision energy was optimised for each MRM transition to achieve the highest possible product ion intensity^22^.

The Norwegian wheat cv. MJO was used as a positive control to develop a SIDA, because it is known to contain the 33-mer^13^. Two different approaches were taken: quantitation of the 33-mer directly in chymotryptically hydrolysed wheat flour and quantitation in hydrolysed gliadins which had been extracted from the flour. The results showed that it was not possible to quantitate the 33-mer directly in hydrolysed flour, because the peptide signal was overlaid by signals originating from the flour matrix. The 33-mer showed signals with high intensity in hydrolysed gliadins and interfering matrix effects were reduced, because only one protein fraction was taken for analysis instead of the entire wheat protein present in flour. To balance out the loss of analyte during sample preparation, the isotopically labelled standard was added prior to chymotryptic digestion of the gliadins.

### Resistance of the 33-mer to enzymatic hydrolysis

Preliminary experiments using a combination of pepsin and trypsin/chymotrypsin (PTC) for enzymatic hydrolysis were performed with the 33-mer. This PTC hydrolysate of the 33-mer was analysed by untargeted LC-MS/MS followed by data evaluation using the MS/MS ions search module of the Mascot software based on the NCBI database (National Library of Medicine, Bethesda, MD, USA). In addition to the original 33-mer, the truncated forms after N-terminal removal of leucine resulting in a 32-mer (*m/z* 950.0, 4+ and *m/z* 1266.3, 3+) and pyro-32-mer (*m/z* 945.7, 4+ and *m/z* 1260.6, 3+) were identified, but no 30-mer (*m/z* 889.7, 4+ and *m/z* 1185.9, 3+) after potential removal of N-terminal LQL. The ratio 33-mer/32-mer/pyro-32-mer was about 12/76/12. To see whether cleavage of the N-terminal leucine could be minimised, only chymotrypsin was used for further experiments. Therefore, the 33-mer peptide was incubated with chymotrypsin using exactly the same conditions as described for the samples to check its resistance towards cleavage, because it contains two potential chymotryptic cleavage sites at positions L1 and L3. In this case, untargeted LC-MS/MS revealed no truncated forms. To ascertain this, full MS/MS scans looking only for the above *m/z* values were acquired and these showed no detectable amounts of 32-mer, pyro-32-mer or 30-mer, only the intact 33-mer. Corresponding experiments, specifically looking for the potential isotopically labelled *32-mer (*m/z* 954.0, 4+ and *m/z* 1271.3, 3+), pyro-*32-mer (*m/z* 949.7, 4+ and *m/z* 1265.6, 3+) and *30-mer (*m/z* 893.4, 4+ and *m/z* 1190.9, 3+), confirmed that the *33-mer standard was also stable under the conditions applied. Full MS/MS scans were also done for the chymotryptic hydrolysates of the albumin/globulin and gliadin fractions obtained from wheat cv. MJO. Again, only the intact 33-mer was detected in the gliadin hydrolysate, but no detectable traces of 33-mer in the albumin/globulin hydrolysate. These experiments confirmed that no truncated forms of the 33-mer were generated during chymotryptic hydrolysis using the applied conditions. This is consistent with the original report by Shan *et al*.^4^ who only observed pepsin-catalysed cleavage of the N-terminal leucine. Additionally, no analyte loss is expected to take place during sample preparation, which involves the removal of albumins/globulins.

### Calibration and quantitation

The response factor of the 33-mer peptide was determined using the peak area ratio A (*33-mer)/A (33-mer) at different values of n (*33-mer)/n (33-mer) between 0.02 and 9.2, that lay within the linear range. Quantitative ^1^H nuclear magnetic resonance spectroscopy (^1^H qNMR) was used to determine the exact concentrations of the methanolic solutions of the 33-mer (1.90 μmol/mL) and the *33-mer standard (1.81 μmol/mL). The characteristic signals located in the aromatic field (δ/ppm: 6.5–8) of the two phenylalanine residues (5 protons each) and the three tyrosine residues (4 protons each) were integrated (22 protons in total) and compared to a reference solution containing L-tyrosine^23^. The area ratio A (*33-mer)/A (33-mer) was obtained from the MS analysis of the MRM transitions *m/z* 987.0 → 279.2 (*33-mer) and *m/z* 979.0 → 263.3 (33-mer). The response factor determined from the slope of the regression line was 0.999. As expected from SIDA, it was very close to 1.0, because analyte and isotopically labelled standard demonstrated the same chemical properties and ionisation behaviour^24^. Quantitation of the 33-mer in all flours was based on this response factor.

### Optimisation of sample preparation

Studies by Fiedler *et al*.^25^ demonstrated that protein digestion was improved with reduction/alkylation in the first step followed by digestion in the second step, because this approach resulted in a higher number of identified peptides. Therefore, reduction of disulphide bonds with tris-(2-carboxyethyl)phosphine (TCEP) followed by iodoacetamide (IDAM) alkylation of liberated free cysteine residues of the gliadin fraction from wheat cv. Akteur (A13) were performed to see if the quantitated amount of 33-mer was influenced compared to native hydrolysed gliadins. After reduction and alkylation, the amount of 33-mer was 6.3 ± 0.4 mg/g gliadin compared to 6.2 ± 0.4 mg/g of native gliadin. The values showed no significant difference (p = 0.705). α-Gliadins typically contain six cysteine residues and form three intrachain disulphide bonds located in the C-terminal domain consisting of sections III, IV, and V^26^. The 33-mer is located within section I and represents a part of the N-terminal domain^27^. After cleavage of disulphide bonds following reduction, the N-terminal domain containing the 33-mer was apparently not affected regarding accessibility to enzymatic attack, which resulted in no significant change of 33-mer contents. To simplify sample preparation, the reduction/alkylation step was omitted.

After chymotryptic hydrolysis of the gliadins extracted from wheat cv. A13, the obtained peptide mixture was purified by solid phase extraction (SPE) using C_18_-cartridges in order to reduce matrix effects. The 33-mer content was 6.3 ± 0.3 mg/g gliadin after purification in comparison to 6.2 ± 0.4 mg/g gliadin without purification of the hydrolysate. The quantitative values were not significantly different (p = 0.506). The only impact was a higher signal intensity (by a factor of 2) of both 33-mer and *33-mer after SPE purification, which did not influence the quantitated amount of 33-mer, because the ratio of analyte to standard did not change. Having ascertained that purification did not influence the content of the 33-mer, the gliadin hydrolysates were analysed by targeted LC-MS/MS without SPE to speed up sample preparation.

### Limit of detection (LOD) and limit of quantitation (LOQ)

The LOD and LOQ of the MS method to quantitate the 33-mer were determined according to Vogelgesang and Haedrich^28^. The analyte was spiked in seven different concentrations between 0.1 and 200 μg/g to rye prolamins as matrix, which did not contain the 33-mer peptide. The absence of the 33-mer in rye flour (cv. Visello) had been confirmed by LC-MS/MS of the hydrolysed flour and prolamin fraction. The 33-mer was identified with high sensitivity resulting in an LOD of 13.1 μg/g rye flour and an LOQ of 47.0 μg/g rye flour.

### Analysis of the 33-mer content in different common wheat and spelt cultivars

The quantitative determination of the 33-mer was performed in flours of 23 hexaploid modern and 15 old common wheat cultivars from different harvest years and two spelt cultivars harvested in 2014 (Table 1). In this context, old common wheat is defined as a cultivar from *T. aestivum* with its year of first registration prior to 1950. All flours were characterised including determination of crude protein contents according to ICC Standard No. 167^29^ and quantitation of α-gliadins, gliadins and glutenins after modified Osborne fractionation combined with RP-HPLC as reported by Wieser *et al*.^30^. Total gluten contents were calculated as sum of gliadin and glutenin contents (see Supplementary Table S1).

**Figure 2:**
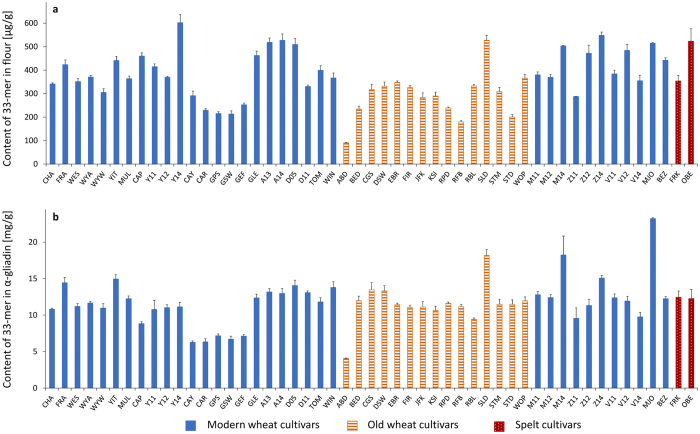
Contents of 33-mer based on flour [μg/g] (**a**) and based on α-gliadins [mg/g] (**b**). 23 modern and 15 old common wheat cultivars (49 samples in total due to multiple harvest years (see Table 1)) and two spelt cultivars were analysed. Wheat cultivars registered prior to 1950 were designated as old. For abbreviations of the cultivars, see Table 1.

The 33-mer was present in all common wheat and spelt flours in a range from 90.9 to 602.6 μg/g of flour (Fig. 2a). The modern wheat Y14 had the highest amount of 33-mer (602.6 μg/g flour), that was significantly different to all analysed cultivars with the exception of Z14 (see Supplementary Table S2). The old wheat ABD with the lowest 33-mer content of 90.9 μg/g flour differed significantly to all wheat and spelt cultivars. In contrast, the old wheat SLD (528.0 μg/g flour) contained one of the highest 33-mer amounts of the analysed flours and showed no significant difference to the modern wheats A13, A14, D05, Z14, V12, and MJO and spelt OBE. Special attention was directed to MJO, because the 33-mer was first identified in this cultivar^13^. The content of 33-mer in MJO (515.0 μg/g flour) showed no significant difference to A13, A14, CAP, D05, GLE, Z12, Z14, V12, and OBE. Most of the modern and old wheat flours contained the 33-mer in a range of 200–400 μg/g flour with an overall average of 368 ± 109 μg/g flour. As a result, only some differences in 33-mer contents between these wheat cultivars were significant. A certain trend, e.g., that modern wheat cultivars generally contain higher amounts of 33-mer than old cultivars could not be derived from the data. Considering the amounts of 33-mer in the two spelt cultivars, it was noticeable that OBE contained one of the highest amounts of the 33-mer peptide (523.4 μg/g flour). The content of 33-mer in FRK (353.9 μg/g flour) was in the range of 200–400 μg/g flour, and did not differ significantly from the common wheat cultivars.

The 33-mer contents of all analysed flours were also calculated based on the amount of α-gliadins (Fig. 2b) determined after modified Osborne fractionation by RP-HPLC^30^. MJO had the highest content of 33-mer in α-gliadin (23.2 mg/g α-gliadin). It was significantly different to all other cultivars (see Supplementary Table S3) and was caused by the high 33-mer content and the low amount of α-gliadins (2.2%) in flour. SLD and M14 had similar 33-mer contents (17–18 mg/g α-gliadin) and differed significantly to all other varieties. ABD had the lowest amount of 33-mer in α-gliadin (4.1 mg/g α-gliadin) and did not show significant differences to CAY, CAR, GPS, GSW, and GEF, but differed statistically to the other cultivars. The overall average content was 11.7 ± 3.1 mg/g α-gliadin.

Many studies in the literature have focused on peptide quantitation in enzymatically hydrolysed prolamin extracts^25^,^31^, hydrolysed gluten extracts^32^ or hydrolysed wheat flours^22^, but the putative immunodominant 33-mer was not quantified. Because of the missing data for 33-mer contents, it was difficult to compare the peptide contents to existing data. Only studies by van den Broeck *et al*. reported the quantitation of the 33-mer using LC-MS with external calibration, but not SIDA. Peptide concentrations were converted into the corresponding contents of α-gliadin per microgram digested gluten protein extract using the average mass of 32,285.5 of α-gliadins. The 33-mer contents determined for two wheat cultivars corresponded to 10.3 and 5.8 mg/g of α-gliadin^33^, which agreed well with the data in Fig. 2b.

### Correlations and principal component analysis

**Figure 3:**
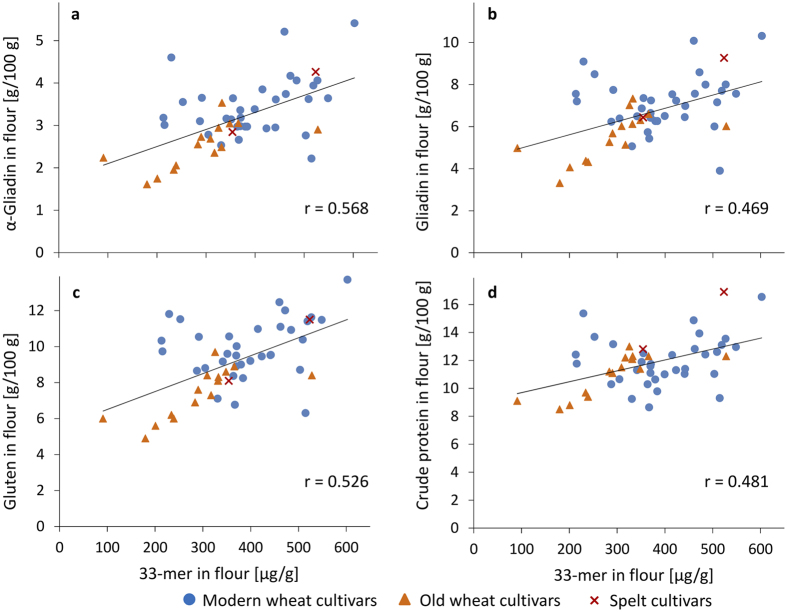
Linear Pearson correlations between contents of 33-mer and contents of α-gliadin (**a**), gliadin (**b**), gluten (**c**), and crude protein (**d**). 23 modern and 15 old common wheat (49 samples in total due to multiple harvest years (see Table 1)) and two spelt cultivars were analysed. Wheat cultivars registered prior to 1950 were designated as old.

The 33-mer contents of the 51 modern and old common wheat and spelt cultivars (based on flour) were correlated to the contents of α-gliadin, total gliadin and total gluten analysed by RP-HPLC after modified Osborne fractionation and to crude protein contents (see Supplementary Table S1). A weak correlation (r = 0.568, p < 0.001) was observed between 33-mer and α-gliadin contents, but there was no correlation to gliadin contents (r = 0.469, p < 0.001), gluten contents (r = 0.526, p < 0.001) or crude protein contents (r = 0.481, p < 0.001) (Fig. 3).

**Figure 4:**
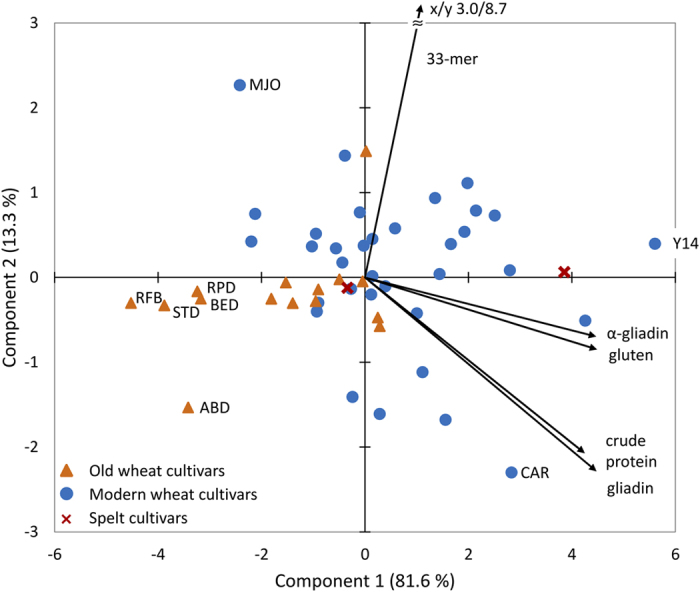
Principal component analysis biplot of data for 33-mer, α-gliadin, gliadin, gluten, and crude protein contents. 23 modern and 15 old common wheat (49 samples in total due to multiple harvest years (see Table 1)) and two spelt cultivars were analysed. Wheat cultivars registered prior to 1950 were designated as old.

Principal component analysis (PCA) with 33-mer, α-gliadin, gliadin, gluten, and crude protein contents of the 49 common wheat and 2 spelt flours was performed to assess whether these variables could be used to differentiate between spelt, modern common wheat, and old common wheat cultivars (Fig. 4). Both principal components together accounted for 94.9% of data variability. Component 1 was positively correlated with 33-mer contents (r = 0.643), but even more so with α-gliadin, gliadin, gluten, and crude protein contents (r ≥ 0.920). In contrast, component 2 was only positively correlated with 33-mer contents (r = 0.765), but negatively associated with α-gliadin, gliadin, gluten, and crude protein contents (r ≤ −0.061). The vector indicating the contribution of the content of 33-mer was downsized for visibility reasons, but it pointed to 3.0/8.7 as x- and y-coordinates. The contents of α-gliadin, gliadin, gluten, and crude protein all had strong positive correlations in all possible pairwise combinations (p ≥ 0.850). PCA essentially confirmed the results of the correlation analyses that had already shown the 33-mer contents to be mostly unrelated to α-gliadin, gliadin, gluten, and crude protein contents. Cv. MJO was placed in the top left corner, because of its high content of 33-mer, but comparatively low contents of gluten proteins, especially α-gliadins. In comparison, cv. Y14 appeared on the far right, because of high contents of all five variables whereas cv. CAR was located in the bottom right corner, because of high (gluten) protein contents, but a comparatively low 33-mer content (229.4 μg/g flour). In addition, PCA revealed that these five variables were unsuitable to differentiate between spelt, modern common wheat, and old common wheat cultivars. The five old common wheat cv. ABD, BED, RFB, RPD, and STD were placed on the far left, but the other ten old cultivars were located right in the middle at similar coordinates as the modern common wheat cultivars. The two spelt cv. FRK and OBE were situated next to the common wheat cultivars. Therefore, the hypothesis that spelt may be less CD-immunoreactive than modern common wheat cultivars could not be confirmed. This finding is in accordance with Ribeiro *et al*.^34^ who compared modern common wheat to spelt cultivars and showed that spelt cultivars had a higher amount of toxic epitopes than common wheats.

### Influence of harvest year and cultivar on the contents of 33-mer

To see whether harvest year or cultivar had a greater influence on 33-mer contents, four wheat cultivars (Mv Magvas, Mv Mazurka, Mv Verbunkos, and Yumai-34) grown at the same location in Hungary (Martonvásár) and harvested in three years (2011, 2012, and 2014)^35^ were studied. The harvest year significantly influenced the 33-mer contents (p < 0.001), whereas the cultivars did not (p = 0.391). There were no significant differences in 33-mer contents between the four cultivars within the harvest year 2011, two out of six differences (V12 vs. M12 and V12 vs. Y12) were significant (p < 0.05) within the harvest year 2012, and three out of six (V14 vs. Z14, V14 vs. Y14, and V14 vs. M14) within the harvest year 2014. Apparently, the environmental factor had a greater influence on 33-mer contents than the genetic background of the four wheat cultivars, because the results for each combination of harvest years (2011 vs. 2012, 2011 vs. 2014 and 2012 vs. 2014) were significantly different (p ≤ 0.034).

### Analysis of durum wheat, emmer and einkorn

The 33-mer peptide was also analysed in two durum wheat and two emmer cultivars (genome AABB) as well as two diploid einkorn cultivars (genome AA) (Table 1). In each of these wheat species, the 33-mer was not detected (<LOD). In comparison to hexaploid common wheat, durum wheat, emmer, and einkorn do not contain the D-genome, which originated from hybridisation of *T. turgidum dicoccum* (genome AABB) with *Aegilops tauschii* (genome DD)^36^. The absence of the 33-mer peptide can be explained by the fact that this peptide is encoded by genes located in the Gli-2 locus on chromosome 6D, which is missing in durum wheat, emmer, and einkorn. Studies by Molberg *et al*. showed clear variations in intestinal T-cell responses between common wheat and tetra- or diploid species due to different degrees of T-cell immunoreactivity between the gluten proteins encoded on the A-, B-, and D-genome. Einkorn cultivars were only recognized by DQ2.5-glia-α1a-specific T-cell clones, but not by DQ2.5-glia-α1b- and DQ2.5-glia-α2-specific T-cell clones. Emmer and durum wheat cultivars were all recognized by DQ2.5-glia-α1a-specific T-cell clones, but only two out of four emmer cultivars and three out of ten durum wheat cultivars activated DQ2.5-glia-α1b- and DQ2.5-glia-α2-specific T-cell clones^37^. Consistent with our results, Prandi *et al*.^38^ found that the 33-mer was not present in durum wheat. As a consequence, this peptide was used as a marker peptide to identify the presence of common wheat in durum wheat flours. One durum wheat cultivar was also analysed by van den Broeck *et al*.^33^ and the 33-mer peptide was not detected either.

## Discussion

The present study is the first to establish a SIDA combined with targeted LC-MS/MS for the quantitative determination of the immunodominant 33-mer peptide in wheat flours. Due to the use of a stable-isotope-labelled *33-mer standard, sample preparation could be simplified without reduction/alkylation and SPE purification.

Although the UniProtKB database had only 19 out of 897 entries for α-gliadin sequences from *Triticinae* containing the 33-mer with an identity of 100%, all 40 analysed modern and old common wheat and spelt cultivars contained the immunodominat 33-mer peptide (51 flour samples in total, because several flours were available from different harvest years). The focus on this peptide seems to be legitimated not only because of its unique structure containing six copies of three overlapping T-cell epitopes, but also because of its presence in all hexaploid wheat cultivars analysed in this study. PCA analysis of the data demonstrated that the contents of 33-mer were not suitable to differentiate old from modern common wheat cultivars, because cultivars with high 33-mer contents were found within both flour sets. The 33-mer was not detected in two cultivars each of tetraploid emmer and durum wheat as well as diploid einkorn, which do not contain the D-genome. This observation may be explained by the fact that the 33-mer is encoded on the Gli-2 locus on chromosome 6D, but a larger set of durum wheat, emmer and einkorn cultivars would have to be analysed to conclude whether these wheat species generally lack the 33-mer peptide. Further work will focus on correlating the content of 33-mer analysed by LC-MS/MS with the gluten content determined by ELISA using the G12 monoclonal antibody.

## Materials and Methods

### Chemicals

The quality of all chemicals was of analytical grade, unless stated otherwise. Disodium hydrogen phosphate dihydrate, ethanol, formic acid (FA; 98–100%), hydrochloric acid (32%, w/w), pentane, 1-propanol, potassium dihydrogen phosphate, sodium chloride, tris(hydroxymethyl)-aminomethane (TRIS), and urea were purchased from Merck (Darmstadt, Germany). IDAM was from Applichem (Darmstadt, Germany). α-Chymotrypsin (from bovine pancreas, TLCK-treated, ≥40 U/mg protein), pepsin (from porcine gastric mucosa, 3200–4500 U/mg protein), TCEP, trifluoroacetic acid (TFA; 99%), and trypsin (from bovine pancreas, TPCK-treated, ≥10 000 BAEE U/mg protein) were obtained from Sigma-Aldrich (Steinheim, Germany), and deuterated methanol-d_4_ containing tetramethylsilane (TMS) was from Euriso-Top (Gif sur Yvette Cedex, France). The peptide LQLQPFPQPQLPYPQPQLPYPQPQPLPYPQPQPF (33-mer) and the isotopically labelled peptide LQLQP*FPQPQLPYPQPQLPYPQPQLPYPQ*PQ*P*F (*33-mer) with *P: L-[^13^C_5_][^15^N]-proline and *F: L-[^13^C_9_][^15^N]-phenylalanine, were purchased from Genscript (Hongkong, PR China) with a purity of >90%. Water for HPLC was purified using an Arium 611VF water purification system (Sartorius, Goettingen, Germany).

### Grain samples

Grains of 23 modern and 15 old (year of first registration before 1950) common wheat cultivars from different harvest years grown worldwide, and one rye cultivar (cv. Visello, harvested 2013, donated by KWS Lochow, Bergen, Germany) were either obtained as flours or milled on a Quadrumat Junior mill (Brabender, Duisburg, Germany) and sieved to a particle size of 0.2 mm. Two spelt, two durum wheat, two emmer, and two einkorn cultivars, grown in Germany (harvested 2014) were milled on a Laboratory 3100 cross beater mill (Perten Instruments, Hamburg, Germany) to wholemeal flours. In total, 57 samples were analysed, because some modern wheat cultivars were available from two to three different harvest years or two cultivation regions (Table 1).

### Characterization of the flours

#### Crude protein content

The crude protein content (nitrogen content × 5.7) of the flours was determined by the Dumas combustion method according to ICC Standard Method 167^29^ using a TruSpec Nitrogen Analyzer (Leco, Kirchheim, Germany).

#### Qualitative and quantitative composition of flour proteins: Osborne fractionation

Flours (100 mg) were extracted with a buffered salt solution (2 × 1.0 mL 0.067 mol/L K_2_HPO_4_/KH_2_PO_4_-buffer, 0.4 mol/L NaCl, pH = 7.6) at 22 °C (room temperature) to obtain albumins and globulins (ALGL). The residues were extracted with ethanol (60%, v/v; 3 × 0.5 mL) at 22 °C (gliadins, GLIA) followed by the glutenin extraction solvent (2 × 1 mL; 50% (v/v) 1-propanol, 0.1 mol/L TRIS-HCl, pH 7.5, 0.06 mol/l (w/v) dithiothreitol) at 60 °C under nitrogen (glutenins, GLUT). After addition of the respective solvent, each flour suspension was vortexed for 2 min and stirred for 10 min (ALGL, GLIA) or 30 min (GLUT). The suspensions were centrifuged for 20 min at 3550 *g* and 22 °C. The corresponding supernatants were combined, diluted to 2 mL with the respective extraction solvent and filtered (WhatmanTM, Spartan 13/0.45 RC, GE Healthcare)^30^.

#### Reversed-phase high-performance liquid chromatography (RP-HPLC)

An UltiMate 3000 HPLC system (Dionex, Idstein, Germany) was used to analyse the extracted ALGL, GLIA, and GLUT fractions. Protein separation was carried out using an Acclaim^TM^ 300 C_18_ column (2.1 × 150 mm, 3 μm, 30 nm, Thermo Fisher Scientific, Braunschweig, Germany). The following conditions were set: solvent A, TFA in water (0.1%, v/v) solvent B, TFA in acetonitrile (0.1%, v/v); linear gradient for ALGL: 0 min 0% B, 0.5 min 20% B, 7 min 60% B, 7.1–11 min 90% B, 11.1–17 min 0% B; linear gradient for GLIA and GLUT: 0 min 0% B, 0.5 min 24% B, 20 min 56% B, 20.1–24.1 min 90% B, 24.2–30 min 0% B; flow rate 0.2 mL/min; temperature, 60 °C; injection volume, 10 μL (GLIA), 20 μL (ALGL, GLUT); detection, UV absorbance at 210 nm. The absorbance areas of PWG-gliadin^39^ (11.6–46.5 μg) were used as calibration standard to calculate the ALGL, GLIA, and GLUT contents of the extracts. The amounts of α-gliadins were calculated from the absorbance area of α-gliadins (retention time 13.5–17.3 min) relative to the total absorbance area of the gliadin fraction. All determinations were done in triplicates.

#### ^1^H qNMR

^1^H qNMR was carried out at 25 °C using a Bruker AV III system (Bruker, Rheinstetten, Germany), equipped with a Z-gradient 5 mm multinuclear observe probe and operated at a frequency of 400.13 MHz. The two peptides (33-mer and *33-mer) were dissolved in methanol-d_4_ and 600 μL of each peptide solution were analysed in 5 × 178 mm NMR tubes (USC tubes, Bruker, Faellanden, Switzerland). The concentrations of the peptide solutions were determined according to Frank *et al*.^23^ using L-tyrosine as reference standard. The signals in the aromatic field were used for integration. ^1^H NMR, δ/ppm (TMS): 6.5–8 (m, 22 H).

#### Sample preparation

First, flours (150–200 mg) were defatted with pentane/ethanol (95/5, v/v; 2 × 2.0 mL)^40^. The gliadin fractions from the different defatted flours were extracted as described above^30^. The gliadin fractions were dried by centrifuging under reduced pressure (40 °C, 6 h, 800 Pa) and re-suspended in a TRIS-HCl-buffer (2.0 mL, 0.1 mol/L TRIS-HCl, pH 7.8, urea 120 mg/mL). The labelled *33-mer peptide was added (300 μL; 10 μg/mL) and the protein-peptide-mixture was hydrolysed with α-chymotrypsin (enzyme-to-protein (E:P) ratio of 1:200) for 24 h at 37 °C. To stop the digestion, TFA (5 μL) was added^41^. The obtained peptide mixture was dried using a vacuum centrifuge (40 °C, 6 h, 800 Pa), re-dissolved in FA (0.1%, v/v, 500 μL), filtered (0.45 μm) and analysed by targeted LC-MS/MS. The same hydrolysis procedure was applied to the 33-mer peptide (10 μg/ml), the *33-mer standard (10 μg/ml) and the albumin/globulin and gliadin fractions of wheat cv. MJO, which were all analysed by untargeted LC-MS/MS. Preliminary experiments were also done with the 33-mer peptide using pepsin (in 0.01 mol/L HCl, pH 2.0, 60 min, 37 °C, E:P ratio of 1:25) followed by trypsin and α-chymotrypsin (in 0.05 mol/L phosphate buffer, pH 6.5, 120 min, 37 °C, E:P ratio of 1:200) according to Dorum *et al*.^8^

### Optimisation of sample preparation

#### Reduction of disulphide bonds and alkylation of cysteine residues

Reduction and alkylation were performed according to Rombouts *et al*.^41^. The extracted gliadin fraction (cv. A13) was re-suspended in a TRIS-HCl-buffer (1600 μL, 0.5 mol/L, pH 8.5) diluted to 50% (v/v) 1-propanol and the reducing agent (TCEP, 0.05 mol/L, 40 μL) was added, followed by incubation for 30 min at 60 °C under nitrogen atmosphere. For alkylation, IDAM was added (0.5 mol/L, 60 μL) and the suspension was incubated for 30 min at 60 °C in the dark. The solution was dried by a vacuum centrifuge (40 °C, 6 h, 800 Pa), the *33-mer was added and the reduced and alkylated gliadins were hydrolysed with α-chymotrypsin and analysed by targeted LC-MS/MS accordingly.

#### Purification of peptides

After hydrolysis, the peptide mixtures were purified by solid phase extraction (SPE) on Supelco DSC-C_18_ tubes (Supelco, Steinheim, Germany). The C_18_-cartridges were conditioned with methanol (1 mL), and equilibrated with TFA (0.1%, v/v, 1 mL). After loading the peptide mixtures, the cartridges were washed with water containing TFA (0.1%, v/v, 5 × 1 mL), and the peptides were eluted with methanol (2 mL). The peptide solution was dried by a vacuum centrifuge (40 °C, 6 h, 800 Pa) and analysed by targeted LC-MS/MS.

#### Untargeted LC-MS/MS

To confirm the resistance of the 33-mer and *33-mer towards chymotryptic cleavage, untargeted LC-MS/MS using an HCTultra PTM ion trap MS (Bruker Daltonics, Bremen, Germany) with collision-induced dissociation (CID) was performed as described in detail by Scherf *et al*.^42^. The untargeted approach was done with standard enhanced scan and auto-MS(n) settings. Additionally, full MS/MS scans of the following precursors were acquired (*m/z* range: target mass ± 1): 32-mer (*m/z* 950.0, 4+, *m/z* 1266.3, 3+), pyro-32-mer (*m/z* 945.7, 4+, *m/z* 1260.6, 3+), 30-mer (*m/z* 889.7, 4+ and *m/z* 1185.9, 3+), *32-mer (*m/z* 954.0, 4+, *m/z* 1271.3, 3+), pyro-*32-mer (*m/z* 949.7, 4+, *m/z* 1265.6, 3+) and *30-mer (*m/z* 893.4, 4+, *m/z* 1190.9, 3+).

#### Targeted LC-MS/MS

A triple-stage quadrupole mass spectrometer (TSQ Vantage, Thermo Fisher Scientific, Dreieich, Germany) was used. The ion source was operated in the ESI positive mode and the following source parameters were set: spray voltage, 4500 V; vaporizer temperature, 50 °C; sheath gas pressure, 40 arbitrary units (au); aux gas pressure, 5 au; capillary temperature, 300 °C. The mass spectrometer was operated in the MRM mode. The most abundant MRM transition was used as quantifier, and the three MRM transitions following in abundance were used as qualifiers. A declustering voltage of −10 V was set for all transitions. The transitions from the precursor ions of the 33-mer and *33-mer to the respective product ions (y-fragments) and the optimised collision energies are shown in Table 2. The 33-mer and the isotopically labelled *33-mer peptides were dissolved in FA (0.1%, v/v, 10 μg/mL). These two stock solutions were mixed in molar ratios n (*33-mer)/n (33-mer) between 9.2 and 0.02 (1 + 9, 1 + 4, 1 + 3, 1 + 1, 3 + 1, 4 + 1, 9 + 1, 14 + 1, 19 + 1, 29 + 1, and 39 + 1) for calibration.

For HPLC separation, an UltiMate 3000 HPLC system (Dionex, Idstein, Germany) was coupled to the mass spectrometer. An XBridge Peptide 3.5 μm BEH-C_18_ column (1.0 × 150 mm, 13 nm; Waters, Eschborn, Germany) was used for peptide seperation. The LC conditions were set as follows: solvent A, FA (0.1%, v/v) in water, solvent B, FA (0.1%, v/v) in acetonitrile; gradient 0–5 min isocratic 5% B, 5–22 min linear 5–55% B, 25–30 min isocratic 90% B; 30–35 min linear 90–5% B, 35–45 min isocratic 5% B, flow rate, 0.1 mL/min; injection volume, 10 μL, column temperature, 22 °C.

#### LOD and LOQ of the MS method

The LOD and LOQ of the quantitation method for the 33-mer peptide were determined. Rye flour (cv. Visello, harvest year 2013) was used as blank, because it was very similar to wheat regarding the gluten protein fractions, but did not contain α-gliadins. The prolamin extraction procedure and chymotryptic hydrolysis were performed as described above. To determine the LOD and LOQ of the targeted LC-MS/MS method, the prolamin extract was spiked at 7 different concentrations (0.1–200 mg/kg) of 33-mer peptide and the samples were hydrolysed by α-chymotrypsin followed by targeted LC-MS/MS analysis. The LOD and LOQ were derived statistically from the data^28^. The LOD was calculated based on a signal-to noise-ratio (S/N) of 3, and the LOQ on an S/N of 10.

### Statistics

Statistically significant differences between 33-mer contents of different modern and old wheat cultivars and two spelt cultivars were determined by one-way analysis of variance (ANOVA) with Tukey’s test as all pairwise multiple comparison procedure at a significance level of p < 0.05 using SigmaPlot 12.0 (Systat Software, San José, CA, USA). The significance of differences between 33-mer contents of the cv. Mv Magvas, Mv Mazurka, Mv Verbunkos, and Yumai-34 harvested in 2011, 2012, and 2014 were analysed by two-way ANOVA accordingly with harvest year and cultivar as factors. Pearson’s product moment correlations were calculated between contents of 33-mer and α-gliadins, gliadins, gluten or crude protein for all analysed wheat and spelt cultivars. Correlation coefficients (r) were defined according to Thanhaeuser *et al*.^43^ (r > 0.78, strong correlation; 0.67–0.78, medium correlation; 0.54–0.66, weak correlation; r < 0.54, no correlation). PCA was carried out with XLStat 2016 (Addinsoft, New York, NY, USA) to determine if the contents of 33-mer, α-gliadin, gliadin, gluten, and crude protein could be used to differentiate between spelt, modern common wheat, and old common wheat cultivars.

## Additional Information

**How to cite this article:** Schalk, K. *et al*. Quantitation of the immunodominant 33-mer peptide from α-gliadin in wheat flours by liquid chromatography tandem mass spectrometry. *Sci. Rep.* **7**, 45092; doi: 10.1038/srep45092 (2017).

**Publisher's note:** Springer Nature remains neutral with regard to jurisdictional claims in published maps and institutional affiliations.

## Supplementary Material

Supplementary Information

## Figures and Tables

**Table 1:** Overview of all 57 samples of modern and old common wheat, spelt, durum wheat, emmer, and einkorn cultivars, their abbreviations, cultivation regions, harvest years, and sources.

Species	Abbreviation	Cultivation region	Harvest year	Source
Cultivar				
**Hexaploid common wheat**				
Chara	CHA	Australia (Victoria)	2014	A
Frame	FRA	Australia (Victoria)	2014	A
Westonia	WES	Australia (Victoria)	2014	A
Wyalkatchem	WYA	Australia (Victoria)	2014	A
	WYW	Australia (West Australia)	2014	A
Yitpi	YIT	Australia (Victoria)	2014	A
Capo	CAP	Austria	2014	A
Mulan	MUL	Austria	2014	A
Carberry	CAY	Canada	2015	A
Cardale	CAR	Canada	2015	A
CDC Go Pen West Seeds	GPS	Canada	2014	A
CDC Go Sara Weigum	GSW	Canada	2014	A
CDC Go Wes Froese	GEF	Canada	2014	A
Glenlea	GLE	Canada	2012	A
Yumai-34	Y11	China	2011	A
	Y12		2012	
	Y14		2014	
Akteur	A13	Germany	2013	A
	A14		2014	
Dekan	D05	Germany	2005	A
	D13		2011	
Tommi	TOM	Germany	2013	A
Winnetou	WIN	Germany	2014	A
Ackermanns Brauner Dickkopf^b^	ABD	Germany	2015	C
Breustedts Extra Dickkopf^b^	BED	Germany	2015	C
Cimbals Großherzog von Sachsen^a^	CGS	Germany	2015	C
Dippes Strum Weizen^c^	DSW	Germany	2015	C
Erbachshofer Braun^c^	EBR	Germany	2015	C
Firlbeck I^c^	FIR	Germany	2015	C
Janetzkis Früher Kreuzung^b^	JFK	Germany	2015	C
Kraffts Siggerländer^b^	KSI	Germany	2015	C
Rimpaus Dickkopf^a^	RPD	Germany	2015	C
Rimpaus Früher Bastard^a^	RFB	Germany	2015	C
Ruppiner Brauner Landweizen^b^	RBL	Germany	2015	C
Steigers Leutewitzer Dickkopf^a^	SLD	Germany	2015	C
Strengs Marschall^c^	STM	Germany	2015	C
Strubes Dickkopf^a^	STD	Germany	2015	C
Walz Oberrheinperle^c^	WOP	Germany	2015	C
**Hexaploid spelt**				
Franckenkorn	FRK	Germany	2014	D
Oberkulmer	OBE	Germany	2014	D
**Tetraploid durum wheat**				
Auradur	AUR	Germany	2014	D
Wintergold	WIG	Germany	2014	D
**Tetraploid emmer**				
Osiris	OSI	Germany	2014	D
Ramses	RAM	Germany	2014	D
**Diploid einkorn**				
Tifi	TIF	Germany	2014	D
Terzino	TER	Germany	2014	D
**Hexaploid common wheat**				
Mv Magvas	M11	Hungary	2011	A
	M12		2012	
	M14		2014	
Mv Mazurka	Z11	Hungary	2011	A
	Z12		2012	
	Z14		2014	
Mv Verbunkos	V11	Hungary	2011	A
	V12		2012	
	V14		2014	
Mjølner	MJO	Norway	2012	B
Bezostaja-1	BEZ	Russia	2012	A

^a^Year of first registration: 1891–1900; ^b^year of first registration: 1901–1910; ^c^year of first registration: 1941–1950; A: MoniQA Association (Monitoring and Quality Assurance in the Total Food Supply Chain, Neutal, Austria); B: kindly provided by Anette Moldestad (Nofima, Ås, Norway); C: kindly provided by Andreas Börner (Leibniz Institute of Plant Genetics and Crop Plant Research, Resources Genetics and Reproduction, Gatersleben, Germany); D: kindly provided by Friedrich Longin (University of Hohenheim, LSA-Research Group Wheat, Stuttgart, Germany).

**Table 2:** Multiple reaction monitoring (MRM) parameters of the 33-mer and the isotopically labelled *33-mer peptides.

Peptide	Precursor ions *m/z* (charge state)	Product ions^1^ *m/z*	Collision energy (V)	Retention time (min)
33-mer	979.0 (4+)^2^1305.2 (3+)^3^	263.3 (y2)^2^	14	19.0
		488.9 (y4)^3^	26	
		713.5 (y6)^3^	14	
		973.5 (y8)^3^	12	
*33-mer	987.0 (4+)^2^1316.0 (3+)^3^	279.0 (y2)^2^	14	19.0
		510.3 (y4)^3^	26	
		735.2 (y6)^3^	14	
		996.0 (y8)^3^	12	

^1^Charge state: 1+.

^2^Precursor to product ion transitions were used as quantifier.

^3^Precursor to product ion transitions were used as qualifier.
