# Supplementary material for: Quantitation of the immunodominant 33-mer peptide from α-gliadin in wheat flours by liquid chromatography tandem mass spectrometry
Source: Sci Rep. 2017 Mar 22;7:45092. doi: 10.1038/srep45092 (PMC5361186; doi:10.1038/srep45092)
Supplement: Supplementary Information [file srep45092-s1.pdf]

Quantitation of the immunodominant 33-mer peptide from α-gliadin in wheat flours by liquid chromatography tandem mass spectrometry

Kathrin Schalk, Christina Lang, Herbert Wieser, Peter Koehler, and Katharina Anne Scherf

Supplementary Table S1.

Contents of α-gliadin, gliadin, gluten, and crude protein [%] in flours of different modern and old common wheat and spelt cultivars.

Wheat cultivar	α-Gliadin [%][1]	Gliadin [%][1]	Gluten [%][1]	Crude protein [%][1]
CHA	3.2 ± 0.0	6.5 ± 0.0	9.2 ± 0.2	11.3 ± 0.1
FRA	2.9 ± 0.1	7.2 ± 0.0	9.5 ± 0.1	11.3 ± 0.0
WES	3.1 ± 0.3	6.9 ± 0.0	9.6 ± 0.4	11.9 ± 0.2
WYA	3.2 ± 0.0	7.2 ± 0.0	10.0 ± 0.0	11.7 ± 0.1
WYW	2.8 ± 0.0	6.4 ± 0.0	8.8 ± 0.0	10.7 ± 0.1
YIT	3.0 ± 0.0	6.5 ± 0.0	9.5 ± 0.1	11.0 ± 0.1
MUL	3.0 ± 0.1	5.7 ± 0.0	8.4 ± 0.0	10.3 ± 0.1
CAP	5.2 ± 0.1	10.1 ± 0.0	12.5 ± 0.1	14.9 ± 0.1
Y11	3.9 ± 0.0	7.5 ± 0.0	11.0 ± 0.0	12.4 ± 0.2
Y12	3.4 ± 0.1	6.6 ± 0.0	9.5 ± 0.0	11.6 ± 0.1
Y14	5.4 ± 0.2	10.3 ± 0.1	13.7 ± 0.2	16.5 ± 0.1
CAY	3.7 ± 0.3	7.7 ± 0.2	10.5 ± 0.1	13.2 ± 0.2
CAR	4.6 ± 0.4	9.1 ± 0.2	11.8 ± 0.4	15.4 ± 0.1
GPS	3.0 ± 0.1	7.2 ± 0.0	9.7 ± 0.0	11.8 ± 0.0
GSW	3.2 ± 0.0	7.6 ± 0.0	10.3 ± 0.1	12.4 ± 0.1
GEF	3.6 ± 0.0	8.5 ± 0.0	11.5 ± 0.0	13.7 ± 0.2
GLE	3.7 ± 0.1	7.6 ± 0.1	11.1 ± 0.2	12.8 ± 0.1
A13	3.9 ± 0.1	7.7 ± .0.3	11.4 ± 0.4	13.1 ± 0.0
A14	4.1 ± 0.0	8.0 ±0.0	11.6 ± 0.0	13.6 ± 0.1
D05	3.6 ± 0.0	7.2 ±0.0	10.4 ± 0.0	12.6 ± 0.1
D11	2.5 ± 0.0	5.1 ±0.0	7.1 ± 0.0	9.2 ± 0.1
TOM	3.4 ± 0.0	6.5 ± 0.1	9.2 ± 0.1	11.0 ± 0.1
WIN	2.7 ± 0.1	5.4 ±0.0	6.8 ± 0.1	8.6 ± 0.1
ABD	2.2 ± 0.4	5.0 ± 0.1	6.0 ± 0.2	9.1 ± 0.1
BED	2.0 ± 0.0	4.4 ± 0.1	6.2 ± 0.1	9.7 ± 0.0
CGS	2.4 ± 0.1	5.1 ± 0.3	7.3 ± 0.5	12.2 ± 1.2
DSW	2.5 ± 0.1	6.1 ± 0.2	8.1 ± 0.2	12.1 ±0.0
EBR	3.0 ± 0.0	6.3 ± 0.0	8.6 ± 0.2	11.4 ± 0.1
FIR	2.9 ± 0.0	7.0 ± 0.3	9.7 ± 0.2	13.0 ± 0.2
JFK	2.6 ± 0.0	5.3 ± 0.0	6.9 ± 0.1	11.2 ± 0.2
KSI	2.7 ± 0.1	5.7 ± 0.0	7.6 ± 0.3	11.1 ± 0.2
RPD	2.1 ± 0.1	4.3 ± 0.3	6.0 ± 0.4	9.4 ± 0.1
RFB	1.6 ± 0.2	3.3 ± 0.1	4.9 ± 0.7	8.5 ± 0.2

RBL	3.5 ± 0.0	7.3 ± 0.0	8.3 ± 0.5	12.3 ± 0.1
SLD	2.9 ± 0.0	6.0 ± 0.1	8.4 ± 0.0	12.3 ± 0.3
STM	2.7 ± 0.3	6.0 ± 0.4	8.4 ± 0.2	11.5 ± 0.0
STD	1.7 ± 0.1	4.1 ± 0.3	5.6 ± 0.4	8.8 ± 0.1
WOP	3.1 ± 0.0	6.6 ± 0.0	8.9 ± 0.3	12.3 ± 0.1
M11	3.0 ± 0.1	6.3 ±0.0	9.0 ± 0.1	10.6 ± 0.2
M12	3.0 ± 0.0	6.4 ± 0.1	8.9 ±0.1	11.1 ± 0.1
M14	2.8 ± 0.0	6.0 ± 0.1	8.7 ± 0.1	11.0 ± 0.1
Z11	3.1 ± 0.0	6.2 ± 0.0	8.7 ± 0.2	10.3 ± 0.1
Z12	4.2 ± 0.1	8.6 ± 0.1	12.0 ± 0.0	13.9 ± 0.2
Z14	3.6 ± 0.1	7.6 ± 0.0	11.5 ± 0.1	13.0 ± 0.1
V11	3.0 ± 0.1	6.3 ± 0.0	8.2 ± 0.0	9.8 ± 0.2
V12	4.1 ± 0.0	8.0 ± 0.0	10.9 ± 0.3	12.4 ± 0.2
V14	3.6 ± 0.0	7.4 ± 0.0	10.6 ± 0.2	12.5 ± 0.0
MJO	2.2 ± 0.0	3.9 ± 0.0	6.3 ± 0.1	9.3 ± 0.1
BEZ	3.6 ± 0.0	7.0 ± 0.0	9.5 ± 0.1	11.4 ± 0.0
FRK	2.8 ± 0.1	6.4 ± 0.3	8.1 ± 0.4	12.8 ± 0.1
OBE	4.3 ± 0.1	9.3 ± 0.1	11.5 ± 0.3	16.9 ± 0.1

[1] mean value ± standard deviation (n = 3) based on flour weight

Supplementary Table S2.

Contents of 33-mer in flours of different modern and old common wheat and spelt cultivars and the significance of differences in 33-mer contents between the 51 flour samples.

Wheat cultivar	33-mer [µg/g][1] in flour	Significant differences[2]
CHA	341.7 ± 4.2	QRSTUVWXYZd
FRA	423.4 ± 20.3	IJKO
WES	351.5 ± 12.2	PQRSTUVWXYZb
WYA	370.7 ± 7.1	OTb
WYW	305.1 ± 16.5	WXYZcek
YIT	441.4 ± 17.3	HIJKN
MUL	363.9 ± 11.3	OZ
CAP	460.2 ± 13.7	DEFGHIJK
Y11	415.3 ± 47.7	JKOP
Y12	370.0 ± 14.1	OU
Y14	602.6 ± 33.4	A
CAY	291.7 ± 6.3	Lmnos
CAR	229.4 ± 19.7	abcdefghijl
GPS	215.6 ± 7.5	pqrs
GSW	213.4 ± 12.9	pqrs
GEF	252.8 ± 7.4	jp
GLE	462.6 ± 18.9	DEFGHIJK
A13	519.0 ± 18.1	BE
A14	527.1 ± 27.2	BC
D05	509.2 ± 26.1	BG
D11	330.9 ± 5.9	RSTUVWXYZg
TOM	399.5 ± 19.6	KOQ
WIN	367.0 ± 21.0	OX
ABD	90.9 ± 1.5	t
BED	234.3 ± 12.0	lmnor
CGS	317.1 ± 23.1	STUVWXYZi
DSW	331.7 ± 18.0	RSTUVWXYZf
EBR	348.3 ± 6.1	QRSTUVWXYZc
FIR	325.5 ± 8.6	RSTUVWXYZh
JFK	283.5 ± 19.6	defghijko
KSI	290.1 ± 16.4	abcdefghijkm
RPD	239.0 ± 3.8	lmnoq
RFB	179.7 ± 5.5	qrs
RBL	332.4 ± 7.0	RSTUVWXYZe
SLD	528.0 ± 22.5	B
STM	309.0 ± 17.9	TUVWXYZj
STD	200.8 ± 10.6	pqrs
WOP	365.6 ± 16.4	OY
M11	380.0 ± 12.8	NOS
M12	369.9 ± 11.9	OV
M14	503.6 ± 1.7	BH
Z11	287.8 ± 43.3	abcdefghijkn
Z12	472.2 ± 34.7	CEFGHIJ
Z14	548.8 ± 13.4	AB
V11	384.0 ± 15.8	MNOR

V12	484.7 ± 25.4	BHI
V14	355.1 ± 22.9	PQRSTUVWXYZa
MJO	515.0 ± 2.7	BF
BEZ	442.4 ± 10.1	HIJKM
FRK	353.9 ± 24.1	OW
OBE	523.4 ± 53.5	BD

[1] Mean value ± standard deviation (n = 3)

[2] Different letters denote significant differences ($p < 0.05$, one-way ANOVA, Tukey's test) between the 33-mer contents

Supplementary Table S3.

Contents of 33-mer in α-gliadin of different modern and old common wheat and spelt cultivars and the significance of differences in 33-mer contents between the 51 flour samples.

Wheat cultivar	33-mer [mg/g][1] in α-gliadin	Significant differences[2]
CHA	10.8 ± 0.1	OPQRSTUVWXZabf
FRA	14.4 ± 0.7	CE
WES	11.2 ± 0.4	LNOPQRSTUVWXZab
WYA	11.7 ± 0.2	HTY
WYW	11.0 ± 0.6	MNOPQRSTUVWZabf
YIT	15.0 ± 0.6	CD
MUL	12.3 ± 0.4	FGHIT
CAP	8.8 ± 0.3	ghi
Y11	10.8 ± 1.2	OPQRSTUVWXZabf
Y12	11.0 ± 0.4	MNOPQRSTUVWXZabf
Y14	11.1 ± 0.6	LMNOPQRSTUVWXad
CAY	6.3 ± 0.2	jk
CAR	6.3 ± 0.4	jk
GPS	7.2 ± 0.2	ij
GSW	6.7 ± 0.4	jk
GEF	7.1 ± 0.2	ik
GLE	12.4 ± 0.5	FGHIQ
A13	13.2 ± 0.5	DEFGHIJ
A14	13.0 ± 0.7	DEFGHIL
D05	14.1 ± 0.7	CF
D11	13.1 ± 0.2	DEFGHIK
TOM	11.8 ± 0.6	HIX
WIN	13.8 ± 0.8	CG
ABD	4.1 ± 0.1	j
BED	12.0 ± 0.6	GHIU
CGS	13.5 ± 1.0	CH
DSW	13.3 ± 0.7	CI
EBR	11.4 ± 0.2	JKLMNPQRSTUVWXZab
FIR	11.1 ± 0.3	MIYf
JFK	11.1 ± 0.8	MNPQRSTUVWXTabe
KSI	10.6 ± 0.6	OPQRSTUVWXZabc
RPD	11.6 ± 0.2	HIZ
RFB	11.1 ± 0.3	LMNOPQRSTUVWXZabc
RBL	9.4 ± 0.2	cdefg
SLD	18.2 ± 0.8	B
STM	11.5 ± 0.7	IYa
STD	11.5 ± 0.6	IYb
WOP	12.0 ± 0.5	GHIV
M11	12.8 ± 0.4	EFGHIN
M12	12.4 ± 0.4	FGHIO
M14	18.2 ± 0.1	B
Z11	9.6 ± 1.4	Zabfh
Z12	11.3 ± 0.8	JKLMNPQRSTUVWXab
Z14	15.1 ± 0.4	C

V11	12.4 ± 0.5	FGHIP
V12	11.9 ± 0.6	GHIW
V14	9.8 ± 0.6	Yzg
MJO	23.2 ± 0.1	A
BEZ	12.3 ± 0.3	FGHIS
FRK	12.4 ± 0.8	DEFGHIM
OBE	12.3 ± 1.25	FGHIR

[1] Mean value ± standard deviation (n = 3)

[2] Different letters denote significant differences ($p < 0.05$, one-way ANOVA, Tukey's test) between the 33-mer contents
